# Effectiveness of a structured education reminiscence-based programme for staff on the quality of life of residents with dementia in long-stay units: A study protocol for a cluster randomised trial

**DOI:** 10.1186/1745-6215-12-41

**Published:** 2011-02-14

**Authors:** Eamon O'Shea, Declan Devane, Kathy Murphy, Adeline Cooney, Dympna Casey, Fionnuala Jordan, Andrew Hunter, Edel Murphy

**Affiliations:** 1 Irish Centre for Social Gerontology, National University of Ireland, Galway, Ireland; 2 School of Nursing and Midwifery, National University of Ireland, Galway, Ireland

## Abstract

**Background:**

Current projections indicate that there will be a significant increase in the number of people with dementia in Ireland, from approximately 40,000 at present to 100,000 by 2036. Psychosocial interventions, such as reminiscence, have the potential to improve the quality of life of people with dementia. However, while reminiscence is used widely in dementia care, its impact on the quality of life of people with dementia remains largely undocumented and there is a need for a robust and fair assessment of its overall effectiveness. The DementiA education programme incorporating REminiscence for Staff study will evaluate the effectiveness of a structured reminiscence-based education programme for care staff on the quality of life of residents with dementia in long-stay units.

**Methods/Design:**

The study is a two-group, single-blind cluster randomised trial conducted in public and private long-stay residential settings in Ireland. Randomisation to control and intervention is at the level of the long-stay residential unit. Sample size calculations suggest that 18 residential units each containing 17 people with dementia are required for randomisation to control and intervention groups to achieve power of at least 80% with alpha levels of 0.05. Each resident in the intervention group is linked with a nurse and care assistant who have taken the structured reminiscence-based education programme. Participants in the control group will receive usual care. The primary outcome is quality of life of residents as measured by the Quality of Life-AD instrument. Secondary outcomes include agitation, depression and carer burden. Blinded outcome assessment is undertaken at baseline and at 18-22 weeks post-randomisation.

**Discussion:**

Trials on reminiscence-based interventions for people with dementia have been scarce and the quality of the information arising from those that have been done has been undermined by methodological problems, particularly in relation to scale and scope. This trial is powered to deliver more credible and durable results. The trial may also convey process utility to a long-stay system in Ireland that has not been geared for education and training, especially in relation to dementia. The results of this trial are applicable to long-stay residential units in Ireland and internationally.

**Trial registration:**

Current Controlled Trials ISRCTN99651465

## Background

Dementia is a chronic progressive debilitating disease that is largely a disorder of old age. It is characterised by widespread impairment of mental functioning, progressive memory loss, language difficulties, confusion and disorientation. These impairments are often accompanied by behavioural and psychological disturbance [1]. The behavioural disturbances associated with dementia are defined as symptoms of disturbed perception, altered thought content, mood and behaviour [2].

Studies on the prevalence of dementia indicate a sharp rise with age, doubling with every 6.3 year increment in age in Western Europe [3]. Current projections indicate that there will be a significant increase in the number of people with dementia in Ireland, from approximately 40,000 at present to 100,000 by 2036 [4]. International estimates suggest that approximately half of those with dementia will be cared for in a residential care setting at some point during the disease [5]. There is limited information on prevalence rates for dementia within long-stay settings, but what is available suggests that the majority of long-stay residents are likely to have dementia. It is estimated, for example, that between 50% and 80% of residents in various types of residential care settings in the UK may have dementia [6]. Recent small-scale estimates for Ireland suggests that a large majority of long-stay residents have cognitive impairment (up to 89%), the majority of whom are likely to have undiagnosed dementia [7]. This data is at variance with official statistics for Ireland, which suggests that only 26% of residents in long-stay care have dementia [8].

The Action Plan for Dementia (APD) for Ireland [9] advocates a person-centred approach to care in both community and long-stay settings that emphasises the individual needs of the person with dementia. Such an approach focuses on what people are still able to do and remember, and on maintaining the individuality of the person with dementia [10]. Promoting a sense of well-being among older people with dementia is enhanced by creating a supportive social environment, thereby enabling people to communicate and connect with family and friends [10,11]. Integrating evidence-based psychosocial approaches with medical and nursing care models of service delivery is key to the person-centred approach. If healthcare professionals are to develop the skills necessary for such an approach to care delivery, education about dementia and staff training in psychosocial treatments are essential [4,9]. Unfortunately, residential care staff in Ireland do not always have the skills and knowledge necessary to respond effectively to the individual needs of residents with dementia [12].

Psychosocial interventions have the potential to improve the quality of life of people with dementia and those who care for them, and a number of systematic reviews have been undertaken [13-15], including Cochrane reviews of specific approaches [16,17]. Reminiscence is a psychosocial intervention commonly used in dementia. It involves the discussion of past activities, events and experiences with another person or group of people, usually with the aid of tangible prompts such as photographs or other familiar items. One factor contributing to the popularity of reminiscence is that it can be used with early memories, which remain relatively intact for people with dementia, thus drawing on the person's preserved abilities rather than focusing on level of impairment induced by the illness. Although used extensively, little is known about the effectiveness of reminiscence as a care intervention for people with dementia [18], [19], [20].

Most studies that have examined the effectiveness of reminiscence have employed qualitative, descriptive or observational designs, with few robust experimental designs having been undertaken [19,21-23]. The most recent Cochrane systematic review of reminiscence therapy in dementia [19] showed that there was evidence of an improvement in cognition and in general behaviour in people with dementia, as well as a decrease in caregiver strain following reminiscence therapy. Five randomised controlled trials were included in this Cochrane review, although only four, with a combined total of 144 participants, had extractable data. The studies were small in scale, incorporating diverse forms of reminiscence therapy, resulting in inconclusive evidence on overall effectiveness. Therefore, the effectiveness of reminiscence on the quality of life of people with dementia remains uncertain, pointing to the need for a more robust and fair assessment of interventions using treatment protocols that set out clearly the type of reminiscence being undertaken, overall objectives, process and outcomes.

Our proposed DementiA education programme incorporating REminiscence for Staff (DARES) intervention is designed to address some of the unanswered questions regarding the effectiveness of reminiscence in the care of people with dementia. The main component of DARES is a structured education reminiscence-based programme for staff which is delivered at the level of the long-stay residential unit to dyad combinations of nursing and care staff who are directly engaged in the care of specified people with dementia.

In this study, we define reminiscence as the deliberate use of prompts, including photographs, smells, music and questioning, to promote the recall of pleasant memories. We view reminiscence as a one-to-one interaction between the person with dementia and a staff member, except where working in a small group is more appropriate, as determined by the capacity and needs of the individual with dementia. Reminiscence is both planned, i.e. where reminiscence is the specific focus of the interaction with the person with dementia, and spontaneous, i.e. the opportunistic use of reminiscence while providing nursing care. The aim of using reminiscence with people with dementia is to stimulate the person, provide enjoyment and foster a sense of achievement and self-worth. The anticipated outcomes for people with dementia of using reminiscence are improvement in the person's quality of life, behaviour and mood.

## Aim

The aim of the DARES study is to evaluate the effectiveness of a structured education reminiscence-based programme for staff on the quality of life of residents with dementia in long-stay residential units. The study has three main objectives:

1. To develop a comprehensive structured education reminiscence-based programme for staff that is specifically orientated toward enabling planned and spontaneous reminiscence to take place as part of the care of people with dementia.

2. To evaluate the impact and effectiveness of the structured education programme within the context of a cluster randomised trial.

3. To understand participants' qualitative perceptions of the education programme, their experience of care following the intervention and its impact on their lives.

## Methods/Design

**Figure 1:**
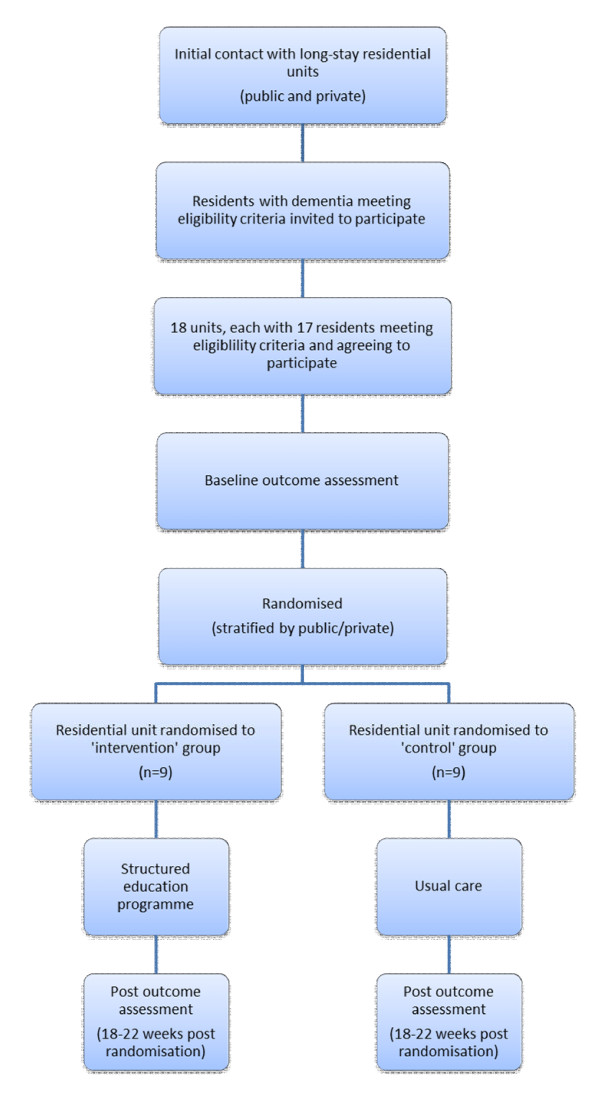
**DARES Methodology**.

The DARES study is a two-group, single-blind cluster randomised trial conducted in public and private long-stay residential settings in Ireland (see Figure 1). Randomisation to control and intervention is at the level of the long-stay residential unit. Care staff within the long-stay residential units allocated to the intervention group receive the structured education reminiscence-based programme. Trained staff use reminiscence with eligible consenting residents within the intervention long-stay settings. Residents in long-stay settings allocated to the control group receive usual care. Blinded outcome assessment is undertaken at baseline and at 18-22 weeks post randomisation. A comparison of outcomes between the intervention and control sites is made to examine if differences exist, and to what extent, between control and experimental groups. Ethical approval has been granted by the Research Ethics Committee of the National University of Ireland, Galway and from each of the appropriate county/hospital-based ethics committees responsible for the public long-stay units in the trial.

We used methods for standard sample size estimates for trials that randomised at the level of the individual [24] adjusting for clustering by inflating sample size estimates by the design effect given by $1+(\bar{n}\text{-1})\rho$, $\bar{n}$ where is the average cluster size, and ρ is the estimated intraclass correlation coefficient (ICC) [25]. Sample size estimates are based on the primary outcome of quality of life of people with Alzheimer's disease as measured by the Quality of Life-Alzheimer's disease (QOL-AD) scale. This instrument consists of two versions; one completed by the person with dementia (care recipient) and the other by the caregiver [26,27]. We chose the care recipient version to estimate sample size for the DARES trial, expressed as the mean rate difference between intervention and control groups.

Based on a mean QOL-AD score of 32.5 for people with dementia in residential care homes [28] and an ICC value of 0.1 identified from pilot work on reminiscence groups for people with dementia for the REMCARE Trial [29], a total of 18 residential units are required, each containing 17 people with dementia, to detect a 4 point difference in mean QOL-AD scores between control and experimental groups, for power of at least 80% with alpha levels of 0.05. This calculation allows for a loss to follow-up of 20% of residents and up to 3 residential units. ICC values lower than 0.1 would increase the power of the study.

### Participants

Public and private long-stay units across the western half of the Republic of Ireland who meet the eligibility criteria were invited to participate in the DARES study. Units are eligible to participate if they have 17 residents with dementia who agree, either directly or through proxy, to take part in the study. This aggregates to a total of 306 residents in the study. Residents are eligible for participation if they have lived in the residential unit for at least one month and are likely to be there for the duration of the study. Given the reality that formal clinical diagnosis of dementia in residential care is rare in Ireland, diagnosis of dementia in residents is determined in any one, or more, of the following ways:

• A formal diagnosis of dementia determined by the DSM-1V or ICD-10 criteria for dementia [30,31]

• Any other diagnosis of dementia by a medical clinician

• Resident is on anti-Alzheimer's medications, including Aricept (donepezil), Ebixa (memantine) and Exelon (rivastigmine)

• Nurses' judgement and/or nursing records advise that the person has dementia

Residents are excluded from the study if they have a significant sensory impairment or an acute physical illness that, in the judgement of the appropriate nursing staff, impairs their ability to participate.

Each nurse and care assistant participating in the study will usually have worked within the care setting for at least three months and be likely to continue working there for the duration of the trial. Five dyads are required to participate in each unit. Each of the participating dyads are allocated three-four participating residents with dementia with whom they implement reminiscence for the duration of the study.

### Randomisation

Randomisation to control and intervention is at the level of the long-stay residential unit. The random allocation sequence is generated using a computer generated random number list (the Mersenne Twister, Stat Direct). Randomisation is 1:1 ratio and is stratified by public and private residential units to ensure an appropriate representation of public and private facilities; a ratio of one third public to two thirds private reflects the overall distribution of beds in the region. Concealment of group allocation is achieved by giving the responsibility for sequence generation and group allocation to a researcher with statistical expertise who is independent of the study and its investigators.

The independent statistical researcher will create a consecutive list of 12 unnamed private units, numbered 1-12, and a separate list of 6 unnamed public units, numbered 1-6. A random allocation sequence is generated and each unnamed unit is assigned a trial allocation based on the sequence. As participatory units agree to enter the trial, the research team will provide the independent statistical researcher with an anonymised list of unit(s) that meet the eligibility criteria. The independent statistical researcher will then document the unique identification code assigned to that unit in the next 'unnamed' position in the randomised public or private list and release the corresponding group allocation for the unit.

### Blinding

This is a single-blind cluster randomised trial. Resident and staff participants within each participating unit are recruited, and baseline data collected, prior to the random allocation of units to control and intervention groups. Because of the nature of the intervention, it is not possible to blind participants (residents with dementia or staff working in the units) to group allocation. Outcome assessment is protected by blinding the research nurses involved in data generation and collection to the group allocation of participating units, staff and residents. Data analysis is undertaken by researchers and statisticians blinded to group allocation by providing a database of outcomes identifiable only by number.

### Intervention

The structured education programme for staff was developed using input from a number of different sources:

• A review of the relevant literature on reminiscence, psychosocial interventions and care for people with dementia

• A concept analysis of reminiscence, undertaken to identify the key attributes of reminiscence

• Interviews with experts from the fields of dementia, dementia education, psychiatry of old age, occupational therapy within psychiatry of old age, psychology and reminiscence

• Interviews with care staff in public and private long-stay units to establish their training needs and preferences for training modalities

• Interviews with people with dementia and with relatives of people with dementia to establish what they feel is important in the care of people with dementia

• A review of standards, guidelines and examples of international best practice on dementia care and dementia education

The education programme is founded on the DARES programme philosophy of empowerment of staff participants, including identifying what participants perceive to be important and what they feel they need to know. The concepts of learner-centeredness and adult learning are at the core of the programme, and those teaching the programme adopt a facilitator-participant role, as this is considered central to realising the empowerment philosophy. Staff participants are trained to enable them to incorporate reminiscence strategies when developing person-centred care plans.

The curriculum incorporates the following sessions:

• Introduction

• Understanding the person with dementia

• How memory works

• Reminiscence explained

• Communicating with persons with dementia

• Behaviours that challenge

• Using reminiscence in practice

• Person-centred care planning for people with dementia

The structured education programme is facilitated by experienced educators and delivered over three days: two consecutive days at the beginning of the intervention and a third day six weeks later. The DARES research team provide telephone support to staff participants during the study period, as well as conducting one support visit to each unit.

Each resident participant in the long-stay unit in the intervention group is linked with a nurse and care assistant who have attended the education programme. Each staff dyad is responsible for using reminiscence with three to four resident participants and for embedding reminiscence within the resident's care plan. Each staff dyad engages the resident with dementia in reminiscence on at least four occasions per week (i.e. one planned formal session and three spontaneous sessions). Staff in the intervention record formal and informal reminiscence conducted with each resident. Staff also complete a life history in order to provide a foundation for the reminiscence sessions.

### Control Group (Usual Care)

Residents in the control group receive usual care where a resident's care is guided by current nursing and medical care plans. This can vary between and within units, but in principle at least, the various elements of care on offer to this group are also available to those in the intervention group. Therefore, the trial examines the additional effects of reminiscence arising from the structured education programme. While the complexity and potential heterogeneity of usual care is acknowledged, every effort is made to describe clearly the components of usual care for residents with dementia through interviews with managers or staff nurses, and through documentary analysis of the residents care plan.

### Consent

The Director of Nursing/Nurse in Charge or their nominee identifies all residents fulfilling the trial inclusion criteria, including the residents' likely ability to participate and the potential benefits or stresses for him/her arising from participation. Residents who would be unduly distressed by inclusion in the study are not asked to consider participation.

Care staff introduce the research nurse (RN) to each potential participant. The RN spends time building a rapport with each potential participant, explains the study in simple language, and explores whether the resident is interested in being included in the trial. If the resident indicates that he/she is not interested in participating in the study, the RN will not pursue consent any further. For residents interested in participating, the RN provides written information about the study. Information about the study is also provided to the relevant next of kin of potential participants. The various information sheets inform potential participants and their families of the purpose, process, potential benefits and harms of the trial, data collection procedures, time commitment, voluntary participation, the right to withdraw (without prejudice to care), as well as providing an assurance of confidentiality.

In seeking consent, it is made clear that participation is voluntary and that the resident has the right to withdraw at any point without prejudice. The resident is given opportunities to indicate how they feel about being involved, to ask questions, clarify issues or to withdraw if they so desire. Where a potential participant is willing to engage in the trial and expresses an understanding of the purpose of the study and its voluntary nature, as well as expressing a choice to participate, the RN will finalise the consent process directly with the resident.

In instances where it is not possible to gain consent directly, consent by proxy is used, where the older person's next of kin is asked to give formal written consent on behalf of their relative. The next of kin is asked to make their decision on the basis of their knowledge of the individual's prior attitudes and values [32]. All next of kin are provided with information on the study, including material on benefits and risks. There is growing evidence that there are differences between the views of people with dementia and their proxies [33] so where possible the perspective of the older person with dementia is sought in the first instance.

Assent is assessed throughout the study for all residents irrespective of whether consent has been obtained directly or by proxy. Where assent is not forthcoming at any stage, the resident is withdrawn from the study without consequence.

## Adverse Events

Reminiscence is this study is used to assist the resident in recalling positive and happy thoughts that enhance communication and connectivity with self and others, thereby improving their quality of life. The risks and harmful side-effects from participating in DARES are, therefore, likely to be low and no adverse reactions were reported from the two pilot sites or from previous trials in the literature [16]. During the structured education programme, staff are trained to deliver reminiscence training that fosters positive thoughts and happy memories. Staff also learn how to respond to situations where reminiscence results in the recall of negative or upsetting events in the lives of residents. Staff must record any such events on the Reminiscence Record Sheets. The research team ask staff during each point of contact (support visit, support telephone calls) whether any adverse events have occurred and offer support as required. If a resident becomes unduly distressed because of reminiscence, staff will respond to the situation in an appropriate manner and if unresolved will raise the issue with the research team. Prospective participants and their families are fully informed of the potential risks and benefits of the project. The resident has the right to opt out of the study at any stage.

## Outcomes

Outcomes are measured for both the control and experimental group at baseline (T_1_) (following consent and prior to randomisation and cluster allocation) and again at 18-22 weeks post randomisation (T_2_).

Each participating long-stay unit is assigned a RN who is responsible for blinded outcome assessment for all participating residents within that unit. All research nurses undertake a two-day preparation programme consisting of:

i) Training on the procedures and protocols surrounding the recruitment of participants and delivery, assessment and recording of all outcome measurements

ii) Simulated completion of all data collection instruments and forms

### Primary Outcome

The primary outcome is quality of life of residents as measured by the Quality of Life-AD (QOL-AD) instrument [26]. The QOL-AD covers 13 domains of quality of life. It is designed to provide both a care recipients (CR) report and a caregiver's (CG) report of the resident's QOL. The measure has good internal consistency, validity and reliability and has been recommended by the European consenus group on outcome measures for use in the measurement of psychosocial interventions in dementia [34].

The QOL-AD is administered as a structured interview using standardised instructions. The RN administers the QOL-AD form to the CR, regardless of the severity of dementia. A staff member who is familiar with the CR completes the caregiver proxy version of the QOL-AD. The RN is available to advise the designated staff member on how to complete the proxy version of the QOL-AD form, answering any questions that may arise in the process.

### Secondary Outcomes

(a) The level of agitation in resident participants is measured using the Cohen-Mansfield Agitation Inventory (CMAI). The CMAI is a 29-item scale specifically developed to assess the frequency of agitated and disruptive behaviours [35]. The questionnaire has four domains: physical/aggressive, physical/non-aggressive, verbal/aggressive, verbal/non-aggressive. The measure has good validity and reliability [36,37]. The CMAI is administered by the RN, following consultation with relevant staff who are aware of the resident's behaviour over the previous two weeks.

(b) Depression in resident participants is assessed using the Cornell Scale for Depression in Dementia (CSDD) [38]. The scale was specifically developed to assess signs and symptoms of major depression in people with dementia across five broad categories. As some people with dementia may not be able to provide reliable reports, the CSDD uses a comprehensive interviewing approach, through two semi-structured interviews: an interview with a main carer and an interview with the person with dementia. Good validity and reliability have been demonstrated for the scale [38].

(c) Staff care burden is assessed using a modified version of the Zarit Burden Interview [39]. The scale was initially developed to assess carer burden on the relatives of impaired older people. The original questionnaire contains 22 questions rated on a 4-point Likert scale and was designed to assess the impact of caring on the physical and emotional well-being of carers. The original scale has satisfactory reliability and validity [40]. A modified version of the original scale is used in this study, containing 13 questions selected for their appropriateness and applicability to nursing care staff in residential long-stay units. The modified scale has performed satisfactorily when used to assess burden on nursing staff caring for people with dementia in a Canadian long-stay setting [41].

## Analyses

### Quantitative Data

The focus is on the long-stay care setting with the resident as the unit of analysis. Analyses is by intention to treat, with all available data included. Quantitative data is analysed, in aggregate, using the Statistical Package for the Social Sciences (SPSS, v17). Data is entered into SPSS, coded and cleaned. Demographic characteristics of staff and of residents are described using percentages, measure of central tendency (means or medians) and measures of variation (standard deviations or ranges). Differences in mean scores are examined using analysis of variance and 2-sample independent t-tests. Relative risks with 95% confidence intervals are calculated with the control group as the reference. Multi-level modelling is used to address the issue of clustering within randomised groups.

Analysis of covariance is used to adjust for baseline differences in outcome variables. Test statistics based on chi-square are divided by the design effect while test statistics based on the t-test or z-tests are divided by the square root of the design effect. Whilst every effort is made to exclude all confounders at the design phase of the study, this may not always be possible. The analysis of data will, therefore, include the search for and control of nuisance variables, for which adjustments had not been made. The change in the primary response variable is modelled across the within subject factors (T_1 _and T_2_), adjusting for explanatory variables as required.

### Qualitative Data

The DARES study includes an embedded qualitative component. This work is done in three parts to:

1) Support the development of the structured education programme. There have been 18 interviews with nurses and health care assistants, 9 interviews with recognised experts in the field of dementia, 3 interviews with people with dementia and 3 interviews with relatives completed.

2) Understand participants' (staff and people with dementia) perceptions of reminiscence, its impact on their lives and their experience of care. There are 9 interviews with nurses, 9 interviews with health care assistants and 9 interviews with people with dementia in the intervention group.

3) Understand and define usual care in the control sites. There are 9 interviews with clinical nurse managers in the control sites.

The primary method of data collection for the qualitative work is in-depth one-to-one interviews, guided by an interview schedule. Contextual data will have already been collected by the RNs during data collection visits to the various sites. This data will help to set the context for the relevant qualitative interviews at these sites. Grounded theory is used to guide the design of the qualitative elements of the study. The constant comparative technique is used to analyse data. Information generated at each data collection point is analysed in full prior to moving to the next stage. This approach enables data analysis to guide ongoing data collection and sampling decisions (theoretical sampling). The qualitative evaluation of residents' experiences of receiving the intervention and their understanding of the intervention will also assist in minimizing threats to fidelity.

## Rigour

Threats to treatment fidelity is minimised by providing the structured education programme within the context of a comprehensive, formal curriculum delivered by experienced educators. Strategies will be put in place to assess the level of reminiscence being conducted in the intervention units, along with remedial action plans where it is found that reminiscence is not being conducted as part of normal care within these units. These strategies include:

- Visiting the unit once between the initial two-day training and the delivery of Day 3

- Using day 3 of the training programme to assess adherence to the programme

- Providing dyads with a telephone number to contact with any queries or problems

- Putting a corrective action plan in place if required for any dyad

Data validation is enhanced by having data collection performed by a small number of trained RNs and by adherence to assessment protocols. Errors are logged by the project manager and remedial strategies implemented as required. The central study processes (e.g. eligibility assessment, outcome assessment etc.) are kept under review to add to the rigour of the study. Single data entry into SPSS is used with visual verification of a sample of records from the data set created from the single entry using a continuous sampling plan (CSP-1) [42].

A pilot study has already been conducted with two residential units, one public and one private. This pilot was used to: identify problems with the research design/processes; refine data collection and analysis; assess adequacy of data sources; examine selection and enrolment processes; test instruments; and assess the resident and staff perspectives on participation in a trial of this complexity. Data from the pilot study is not included in the main analyses of the trial.

## Discussion

The 2009 World Alzheimer Report [3] contains a beautiful picture of an older woman called Jacqueline taking part in a reminiscence therapy session in Nice, France in 2008. The woman is relaxed and smiling, obviously enjoying whatever happy memories are being evoked during the session. Another picture, in the same publication, shows a woman with dementia, a former mathematics teacher, writing numbers on a blackboard. The latter was purchased by care staff to help the woman feel connected to her past and allow her the opportunity to experience old pleasures. These pictures embody the belief that reminiscence-based care has the potential to enhance the quality of life of people with dementia in a variety of care settings.

The problem is that we know relatively little about the overall effectiveness of reminiscence as a care intervention, with a recent Cochrane review concluding that there was an urgent need for more quality research on the impact of reminiscence-based care on residents with dementia and care staff. Reminiscence itself has diverse roots, ranging from psychotherapy (the life review), involving sometimes painful evaluation of personal memories, to oral history, which has the simpler aim of enhancing communication and connectivity in an enjoyable engaging fashion. Reminiscence, as used in this study, focuses on the latter through discussion of past activities, events and experiences, usually with aid of familiar items from the past to prompt memory, making use of the cognitive strengths of the person with dementia rather than any cognitive weakness. Many older people with dementia suffer from reduced psychological well-being and reminiscence has potentially a lot to offer them, particularly in relation to maintaining identity and a more complete realisation of the self. It also may assist carers in developing a deeper attachment and connection to the person with dementia, thereby enhancing personhood and the whole caring experience.

This study will report on an on-going large trial that will examine the effect of a reminiscence-focused dementia education programme for care staff on quality of life, agitation and depression for people with dementia living in long-stay units in Ireland. Staff attitudes to residents with dementia and perceived care burden among staff are also measured. The study has the potential to shed light on key measurement and methodological issues that arise when conducting psychosocial trials of this nature on people with dementia in long-stay settings. It will also provide evidence on the usefulness of reminiscence-based education programmes for staff as a means of orienting care practice towards more person-centred care.

## Competing interests

The authors declare that they have no competing interests.

## Authors' contributions

EOS, KM, DC, AC, FJ, AH, EM and DD designed the trial protocol and secured funding for the DARES Trial. EOS and DD drafted the manuscript and KM, DC, AC, FJ, AH, EM contributed to the manuscript. All authors read and approved the final manuscript.
